# Understanding the real-time interaction between middle-aged consumers and online experts based on the COM-B model

**DOI:** 10.1057/s41270-022-00196-1

**Published:** 2022-11-23

**Authors:** Lifu Li, Kyeong Kang

**Affiliations:** Faculty of Engineering and Information Technology, University of Technology Sydney, Sydney, NSW Australia

**Keywords:** Live streaming shopping, Middle-aged Chinese consumers, Online experts, Counterfeiting concern, COM-B model

## Abstract

This paper presents a study of middle-aged online consumers’ specific shopping behaviour on live streaming platforms and analyses the distinct marketing strategy provided by online experts. Influenced by unique social and cultural backgrounds, middle-aged online consumers lack related shopping experience and keep counterfeiting concerns to live streaming shopping, making them prefer to interact with online experts before making final decisions. Based on the COM-B Behaviour Changing theory and the Emotional attachment theory, the research model has been established in this study, and it divides influencing factors into the *Emotion* unit, O*pportunity* unit and *Capability* unit. To test the relationships between influencing factors and middle-aged online consumers’ interactive motivation, the partial least-squares path modelling and variance-based structural equation modelling (PLS-SEM) have been applied on the SmartPLS. By analysing 450 samples, the study shows that the counterfeiting concern and ease of use factors positively impact online consumers’ motivation to interact with online experts, and self-efficacy plays a negative role.

## Introduction

Unlike the traditional online shopping model, live streaming shopping established on live streaming platforms (LSPs) can help online consumers reach a broad market, purchase products at reduced costs and interact with sellers in real time (Schaupp and Bélanger 2019; Johnson and Woodcock 2019). According to the definition proposed by Johnson and Woodcock (2019), LSPs are websites or applications that are developed based on peer-to-peer technology and enable users to broadcast live videos. The live streaming content can be sent over the platform in real time without first being recorded. Among these LSPs in China, TikTok (Douyin) is the most popular one, and it has attracted more than 500 million active users and has become the third most downloaded app worldwide (Chaffey 2019; Zhou 2019). Similar to the TikTok platform, the number of users from other LSPs, like Twitch, YouTube Live, Kuaishou, Jingdong Live and Taobao Live, has also increased dramatically in recent years (Li et al., 2022a, b). According to the Chinese live streaming market report (2019c), from 2018 to 2019, the number of active LSP users has increased dramatically from 230 to 330 million, and 63.1% of them claim they pay attention to live shopping information.

During live streaming, some sellers introduce their products themselves, and some may design a unique marketing strategy and invite online experts to attract online consumers’ attention (Wu and Yuan 2019). According to the definition presented by Lin et al. (2018), online experts are online opinion leaders with deep knowledge and skills in a specific area, and they have high credibility to present professional content on their live streaming channels (Lin et al. 2018). In detail, they often demonstrate professionalism by showing their professional certificates and degree titles during live streaming. However, due to the lack of a sound supervision system, live shopping transaction-related issues would happen during live streaming shopping, such as false advertising and misleading information provided by online experts (Liu 2022; Liu et al. 2020). This results in some counterfeit and shoddy products being recommended by online experts, which would harm online consumers’ live shopping experience, especially middle-aged Chinese online consumers.

Middle-aged Chinese online consumers are the consumer group that is influenced by unique social and cultural backgrounds, and they are between 40 and 60 years old. Most of them could have specific shopping behaviours on LSPs, such as keeping counterfeiting concerns and preferring to follow formal suggestions (Qian et al. 2019; Tong et al. 2020). For instance, to avoid uncertainty and risk, middle-aged Chinese online consumers tend to build trust with online experts who have professional certificates (Lu et al. 2018; Liao et al. 2017). Hence, they are willing to interact with online experts rather than typical live streamers. Meanwhile, although live streaming shopping has been promoted on various LSPs since 2017, some middle-aged online consumers still are unfamiliar with this new online shopping mode, causing their purchasing intention would potentially be influenced by online experts (Chen et al. 2020a; Lv and Luo 2018; Sun et al. 2019). In light of this, it is significant for this study to consider the middle-aged Chinese online consumers’ social and cultural backgrounds and analyse what factors would have essential impacts on their motivation to interact with online experts.

Faced with these potential issues, two research questions have been presented in this paper. First, the development of LSPs provides middle-aged Chinese online consumers with various convenient functions to interact with online experts, such as real-time video, fans group chat and virtual gift-sending system (Schaupp and Bélanger 2019). Unlike traditional online shopping platforms, the ease of use of LSPs narrows the distance between online consumers and experts, decreasing online consumers’ engagement difficulties. In addition to the ease of use factor, middle-aged online consumers’ personal capability, also known as self-efficacy, would affect their motivation to interact with online experts (Gao 2018). According to the survey promoted by Lu et al. (2018) and Gao (2018), most middle-aged online consumers have less than 1 year of live streaming using experience and are unfamiliar with online experts’ marketing strategies. This leads them to pay more attention to the product price rather than other product details. Unlike other age groups, a weaker sense of self-efficacy drives middle-aged consumers to blindly rely on experts’ recommendations, probably causing them to buy counterfeit products. Thus, the first research question is *How do LSPs’ ease of use and self-efficacy factors affect middle-aged Chinese online consumers’ motivation to interact with online experts?*

To solve the first question, this paper draws on the COM-B Behaviour Changing theory to discover why middle-aged Chinese consumers tend to interact and build trust with online experts (Michie et al. 2011). According to the author Michie et al.’s research (2011), individuals’ motivation is influenced by *Opportunity* and *Capability*, and individuals’ behaviour is directly influenced by their motivation. To be specific, the *Opportunity* can be combined with the LSPs’ ease of use. *Capability* can be designed as the middle-aged consumers’ self-efficacy. In addition to the COM-B Behaviour Changing theory, the research also refers to the Emotional attachment theory based on consumer psychology research, and it pays much attention to personal *Emotion* factor, i.e. counterfeiting concern (Marcketti and Shelley 2009; Ahmed et al. 2017; Bian and Haque 2020). According to the definition presented by Ahmed et al. (2017), counterfeiting concern is an emotional factor existing in online consumers’ thinking and plays a negative role in the security and reliability of live shopping systems. Although existing literature applies the COM-B model to explore online consumers’ behaviours from macro and micro aspects, almost none of them improves the COM-B Behaviour Changing model and adds the *Emotion* unit based on the Emotional attachment theory (Lu et al. 2018; Liao et al. 2017; Li and Kang, 2021b; Li et al., 2021). Thus, considering the effect of counterfeiting concern, the paper's second research question is *How does the counterfeiting concern affect middle-aged Chinese online consumers’ motivation to interact with online experts?*

This paper has both theoretical and practical implications based on the research results. Regarding the theoretical contribution, the study utilises the COM-B Behaviour Changing theory to design the research model. It also analyses how influencing factors from *Opportunity* and *Capability* units affect middle-aged Chinese online consumers’ motivation to interact and build trust with online experts. In addition to the COM-B model, this study also considers the *Emotion* unit, i.e. counterfeiting concern factor, from the Emotional attachment theory to improve the research model. This is beneficial to conform to the particular live shopping behaviours of middle-aged Chinese online consumers. Meanwhile, this paper explores the interaction process between middle-aged online consumers and online experts, which extends online consumer behaviour research based on the systematic research model. According to the analysis results, the advice related to enhancing middle-aged online consumers’ live shopping awareness and improving the live streaming market environment will be shown in the practical implications part.

## Literature review

### Middle-aged consumers

Individuals’ specific behavioural response is influenced by particular social and cultural backgrounds, which can be utilised to analyse middle-aged Chinese online consumers’ live shopping behaviours (Ying et al. 2021). Most previous studies focus on potential issues in online shopping, such as false online product reviews and exaggerated advertising problems (de Luna et al. 2020; Sharma et al. 2019; Xu et al. 2020a). Some discover how online consumers’ purchase intention is influenced by the interaction with live streamers (Cai and Wohn 2019; Chen et al. 2019; Wongkitrungrueng and Assarut 2018). However, almost no researchers focus on middle-aged Chinese online consumers and explore why they tend to interact with online experts rather than typical live streamers on LSPs. Unlike prior studies, this paper analyses middle-aged Chinese online consumers aged between 40 and 60 years old. They have unique live shopping behaviours that differ from other age groups. Firstly, although middle-aged Chinese consumers’ income levels are significantly higher than the younger and older groups, many still keep cautious thinking during live streaming shopping, which can be explained based on the traditional Chinese conservatism culture (Soon and Liu 2020). Influenced by the traditional Chinese conservatism culture, middle-aged online consumers tend to think twice before making a final decision. Secondly, most live shopping consumers are from the younger group (between 20 and 39 years old), but few middle-aged online consumers are familiar with this new shopping mode, causing the lack of a comprehensive understanding of live streaming shopping (Park and Lin 2020). Unlike offline shopping, middle-aged online consumers have to control live shopping skills, such as product search and quality comparison. Due to the limited live streaming shopping experience, they prefer to seek online experts’ advice rather than making decisions alone (Li and Kang 2020). Thirdly, most middle-aged consumers have been born before China’s economic reform and experienced economic slack or inefficiency (Lau and Zheng 2017). The backward economic environment and unbalanced development between urban and rural areas might increase financial pressure and foster risk aversion thinking (Long et al. 2016; Bilby et al. 2020). Thus, compared with other age groups consumers, middle-aged Chinese online consumers would be more cautious during the live shopping process, reflecting on their counterfeiting concern. The emotional attachment could motivate them to interact with online experts instead of typical live streamers.

### Emotion

The Emotional attachment theory claims that consumers’ emotional attachment to an object could predict their interaction with it (Kaufmann et al. 2016). Based on the theory, counterfeiting concern as an emotional factor exists in online consumers’ thinking and plays a significant role in the security and reliability of live shopping systems (Ahmed et al. 2017). Previous scholars refer to the Emotional attachment theory and apply the *Emotion* factor named counterfeiting concern to the study of online consumers’ shopping behaviour (Marcketti and Shelley 2009; Kim 2019; Bilby et al. 2020; Bian and Haque 2020; Kaufmann et al. 2016). However, almost none of them evaluates the counterfeiting concern impact on middle-aged Chinese online consumers. Considering middle-aged Chinese consumers’ particular social and cultural backgrounds, counterfeiting concern would significantly impact their live shopping behaviour and stimulate them to interact with online experts. Specifically, different from other age groups, middle-aged Chinese consumers hold dependent thinking and prefer to seek advice from others to avoid unnecessary risks. This kind of thinking style could give them a sense of security and strengthen their relationship with online experts during live shopping (Ji et al. 2017). Meanwhile, due to experiencing the difficult period when the economic system is unimproved, middle-aged online consumers are more likely to produce counterfeiting concerns during live streaming shopping than younger consumers (Li and Kang 2020, 2021a). In their opinion, the counterfeiting concern can help them reduce financial losses and avoid unnecessary trouble. Therefore, this study focuses on the impact of counterfeiting concerns and applies its emotional influence to explore why middle-aged online consumers tend to interact with online experts.

### Opportunity and capability

According to the COM-B Behaviour Changing theory established by Michie et al. (2011), both *Opportunity* and *Capability* factors affect individuals’ motivation, leading to final behaviour. As an *Opportunity* factor, ease of use refers to the degree to which a consumer believes that using the live streaming system would be free of effort (Cai et al. 2018). Specifically, the technical development of LSPs provides middle-aged Chinese consumers with a convenient technical opportunity to interact with online experts in real time (Li and Kang 2020). Up to December 2018, the number of Chinese live streaming users has increased to 829 million, and most of them claim that the attractive factor of live streaming shopping is the ease of use (2019a; Chen et al. 2020b). With the improvement of peer-to-peer technology in China, various functions on LSPs, such as real-time group chat, virtual gift-sending system and online store functions, provide online consumers with a comfortable opportunity to communicate with online experts (Schaupp and Bélanger 2019; Lu et al. 2018). Thus, ease of use is an essential factor that should be focused on when analysing the interaction between middle-aged consumers and online experts.

According to the definition proposed by Li et al. (2018), self-efficacy is online consumers’ belief that they have the capability to perform a particular behaviour, and consumers with high self-efficacy have confidence in their ability. For the current study, self-efficacy refers to the ability of middle-aged consumers to distinguish the authenticity of products recommended by online experts. As a *Capability* factor, it would affect middle-aged online consumers’ motivation to interact with online experts on LSPs. Although the Chinese government have promoted economic reform since 1978, and the living standard of Chinese people has improved dramatically, many middle-aged groups still lack comprehensive education and are not familiar with the new shopping mode, reflecting on their self-efficacy (Han and Ye 2017; Li et al. 2018). Middle-aged Chinese online consumers with low self-efficacy would prefer to build trust with online experts rather than evaluate the quality of the product by themselves (Lu et al. 2018). As Table 1 shows, the certification presented by the jade expert on the TikTok platform has strong attractiveness for middle-aged consumers, and they are willing to purchase the jewellery recommended by the expert. Meanwhile, online experts have more marketing skills and more comprehensive expertise than typical live streamers. Due to the lack of live shopping experience and skills, middle-aged online consumers’ shopping interest would be influenced by online experts who use wildly exaggerated, fanciful, or vague claims for a product. Although some consumers have the capability to distinguish exaggerated advertisements, their rational judgement of products would be inflated (Virdi 2021). Thus, middle-aged online consumers’ self-efficacy would negatively influence their motivation to interact with online experts and build trust with them.

**Table 1:** The example of online experts on LSPs

	

## Research model and hypotheses

**Fig. 1:**
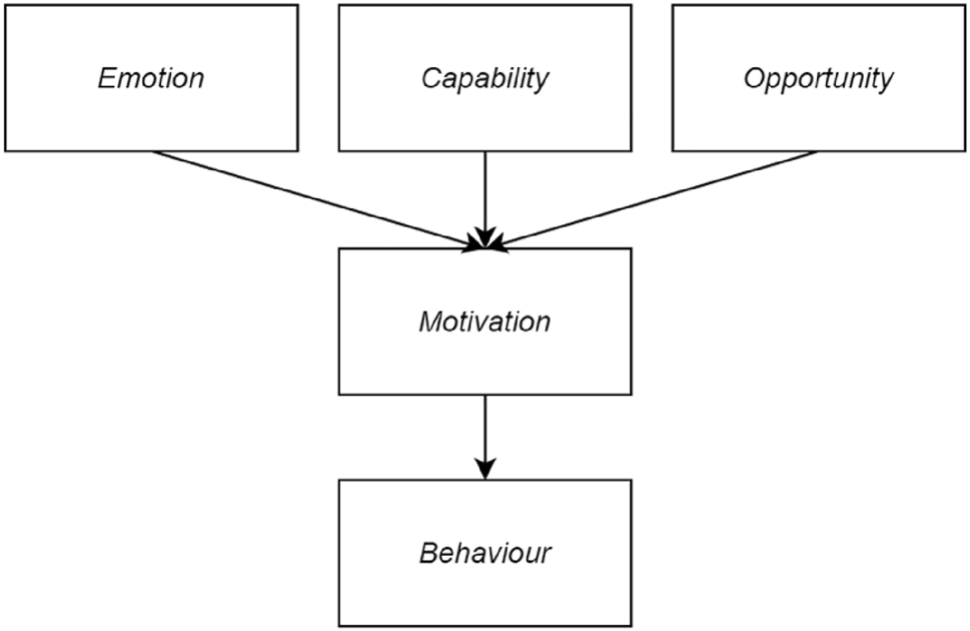
The COM-B behaviour change model

**Fig. 2:**
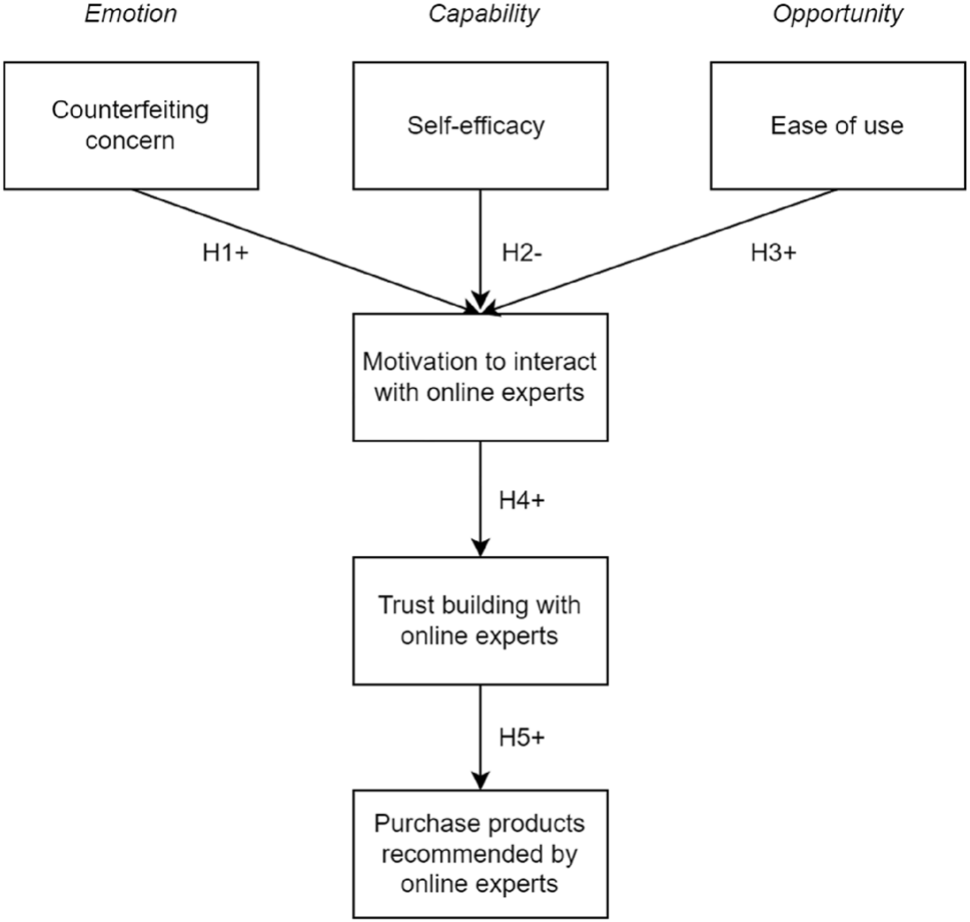
The research model

To analyse the interactive process between middle-aged Chinese online consumers and online experts, this paper applies the COM-B Behaviour Changing model that includes two independent units, *Opportunity* and *Capability*, as Fig. 1 shows (Michie et al. 2011). Drawing on Michie et al.’s research (2011), the ease of use of LSPs belongs to the *Opportunity* unit, and middle-aged online consumers’ self-efficacy can be designed into the *Capability* unit. *Motivation* refers to a quantity attached to final behaviours, and it could be affected by *Capability* and *Opportunity*. Hence, online consumers’ motivation to interact with online experts would drive these consumers to build trust with online experts, leading to their final purchase behaviours. Meanwhile, the research model also draws on the Emotional attachment theory and designs the *Emotion* factor, i.e. counterfeiting concern, in the study of middle-aged consumers’ shopping behaviour (Marcketti and Shelley 2009; Kim 2019; Bilby et al. 2020; Bian and Haque 2020), which is suitable for the study promoted under Chinese specific social and cultural backgrounds. Furthermore, *Behaviour* is the result of an interaction between three components, aiming to identify what actions consumers could be displayed (Michie et al. 2011). Therefore, this research model has been established, as Fig. 2 shows.

### Effect of emotion unit

Influenced by China’s particular social and cultural backgrounds, middle-aged online consumers tend to keep counterfeiting concerns during live streaming shopping, aiming to avoid unnecessary issues, such as counterfeit products and fake advertising (Soon and Liu 2020; Xing 1995). Specifically, as a developing country, most middle-aged Chinese online consumers have been born before China’s economic reform and experienced financial burden, resulting in their lack of confidence during live streaming shopping (Bilby et al. 2020; Yao et al. 2011; Lau and Zheng 2017). Meanwhile, unlike other age groups, middle-aged Chinese online consumers are willing to seek advice from others before making a final decision, which fosters them to interact with online experts (Ji et al. 2017). Thus, considering middle-aged Chinese consumers’ particular social and cultural backgrounds, the counterfeiting concern factor would significantly impact their live shopping behaviour and stimulate them to interact with online experts. Therefore, the paper proposes the following hypothesis:

#### Hypothesis 1

The counterfeiting concern positively affects middle-aged Chinese online consumers’ motivation to interact with online experts during live streaming shopping.

### Effect of capability unit

Due to the lack of live shopping experience, middle-aged Chinese online consumers would consider risk aversion and prefer to follow online experts’ suggestions rather than typical live streamers (Lu et al. 2018; Huang, 2013). Although some experts may make exaggerated and false advertising, their professional certificates and titles could still attract many middle-aged online consumers to their live streaming channels (Ji et al. 2017). Meanwhile, although the living standard of Chinese people has improved dramatically, many middle-aged groups still lack comprehensive education and are not familiar with the new shopping mode, decreasing their self-efficacy (Han and Ye 2017; Li et al. 2018). To enhance product sales, online experts could utilise various marketing strategies and advertising skills to affect online consumers’ rational attitude to live shopping (Liu et al. 2016). This means that middle-aged online consumers with low self-efficacy would be easily attracted by online experts. Thus, the paper proposes:

#### Hypothesis 2

The self-efficacy negatively affects middle-aged Chinese online consumers’ motivation to interact with online experts during live streaming shopping.

### Effect of opportunity unit

Different from traditional social media platforms, the ease of use of LSPs, such as convenient functions and comprehensive services, provides middle-aged online consumers with comfortable chances to interact with online experts, which has been identified by prior research (Schaupp and Bélanger 2019; Lu et al. 2018). As peer-to-peer technology improves in China, various improved functions on LSPs, like real-time video, online store display and fan group service, ensure that the interaction between online consumers and online experts is smooth and efficient (2019a; Chen et al. 2020b). Therefore, the ease of use of LSPs provides middle-aged Chinese online consumers with a comfortable opportunity to interact with online experts, and the paper hypothesises:

#### Hypothesis 3

The ease of use positively affects middle-aged Chinese online consumers’ motivation to interact with online experts during live streaming shopping.

### Motivation and final behaviour

According to China’s live streaming industry research report (2019b), more than 93.2% of online consumers choose live streaming shopping because they can have real-time interaction with live streamers and communicate product details with online experts. Meanwhile, suitable interaction content, such as satisfaction guarantees, returns and refund policies, could also have a favourable impact on middle-aged online consumers’ attitudes to products and win their trust (Oliveira et al. 2017). Thus, the paper supposes:

#### Hypothesis 4

The motivation to interact with online experts positively affects middle-aged Chinese online consumers to build trust with online experts during live streaming shopping.

As discussed before, compared with other age groups, middle-aged Chinese online consumers are more comfortable building trust with online experts and purchasing the online products they recommended (Lu et al. 2018; Alvarado et al. 2020). Moreover, online experts understand the importance of presenting professional certificates, and they utilise their reputation to attract middle-aged online consumers’ attention to increase their sales. Therefore, once online consumers build trust with online experts, their final purchasing behaviours will be influenced. The paper proposes:

#### Hypothesis 5

Trust building with online experts positively affects middle-aged Chinese online consumers to purchase the products recommended by online experts during live streaming shopping.

## Methodology

### Research setting

An online survey has been conducted in this study to evaluate the research model. Compared with offline research methods, the online survey method has the advantage of collecting data from various user groups coming from different areas, especially during the COVID-19 pandemic (Nayak and Narayan 2019). The study utilises middle-aged Chinese online consumers as samples and distributes online questionnaires among them. This not only considers the rapid development of China's live streaming market but also because online experts are prevalent in China's live streaming shopping. In detail, the number of active live streaming users has increased dramatically from 230 to 330 million from 2018 to 2019, and more than 63% of them focused on live shopping information (2019c). Based on the rapid development of live streaming commerce and the vast number of middle-aged online consumers, the Chinese live shopping environment is chosen as the research context.

### Measurement

All constructs measured in this paper are designed according to previous studies. The counterfeiting concern is estimated based on three questions from Marcketti and Shelley’s consumer behaviour research (2009). Middle-aged online consumers’ self-efficacy and ease of use of LSPs are assessed by the study of Pavlou and Fygenson (2006). Drawing on Lim et al.’s paper (2012), three questions are updated to evaluate middle-aged online consumers’ motivation to interact with online experts. Adapted from Bart et al.’s online consumer survey (2005), middle-aged Chinese online consumers’ trust building with online experts is tested through three questions. Finally, according to the previous papers related to online consumers’ planned behaviours, three-question items about online consumers’ final purchasing behaviours are applied in this paper (Pavlou and Fygenson 2006). In addition to basic information statistics, the main questionnaire content is shown in Table 2. To assist participants in answering each question more accurately, the paper utilises the Likert 7-point scale with a range from the lowest score = 1 to the highest score = 7 (Dawes 2008).

**Table 2:** The list of questionnaire contents

Variable	Item	Measurement
Counterfeiting concern Marcketti and Shelley (2009)	CC1	How concerned are you about product issues such as counterfeiting?
	CC2	How concerned are you about counterfeiting?
	CC3	How concerned are you about the effects of counterfeit apparel when making purchases?
Self-efficacy Pavlou and Fygenson (2006)	SE1	If I wanted to, I could become skilful at comparing and evaluating products on the live streaming platform
	SE2	If I wanted to, I could quickly become knowledgeable about getting all relevant streamers and products from live streaming platforms
	SE3	I have abundant experience using the live streaming platform and interacting with online experts
Ease of use Pavlou and Fygenson (2006)	EU1	Communicating with online experts about the related product from the live streaming platform would be easy for me
	EU2	Interacting with online experts and purchasing products are easy for me
	EU3	Learning how to use live streaming platforms would be easy
Motivation to interact with online experts Lim et al. (2012)	IO1	I feel I usually motivate to interact with online experts
	IO2	I am willing to participate in the interaction with online experts
	IO3	Interacting with online experts on the live streaming platform is vital for me
Trust building with online experts Bart et al. (2005)	TB1	The online experts in the live streaming platform appear to be more trustworthy than other live streamers
	TB2	My overall trust in online experts is high
	TB3	I have confidence in online experts’ recommendations
Purchase products recommended by online experts Pavlou and Fygenson (2006)	PP1	I intend to purchase the products recommended by online experts
	PP2	I plan to purchase the products sold by online experts
	PP3	I am willing to purchase the product introduced by online experts

### Data collection

Academic design functions and multilingual options on the questionnaire platform named Tencent Questionnaire are suitable for middle-aged Chinese online consumers to fill in, which has been identified by prior studies (Xu et al. 2020b). Considering the Chinese language environment, the online questionnaire content has been translated into Chinese by scholars who are skillful in English and Chinese languages. To focus on the target respondents who are both familiar with live streaming shopping and online experts, many filtering questions have been designed before the formal questionnaire, such as participants’ age, platform-using experiences and interactive experiences with online experts. Before middle-aged participants fill in online questionnaires, the invitation letter is distributed in advance to assist them in understanding the research topic. From July 2021 to August 2021, 526 questionnaires are collected from 28 different provinces. Among these 526 questionnaires, inappropriate responses have been deleted, including the same responses, same IP address, incomplete responses and unfamiliar with online experts’ live streaming. Finally, 450 questionnaires are valid for this research, and the return rate is 85.55%.

## Data analysis

### Descriptive statistics

Among these 450 respondents (Table 3), 50.89% of them are female, and 49.11% are male. More than 32% of them are between 46 and 50 years old, 25.56% of them are between 40 and 45 years old, and 24.22% are between 51 and 55 years old. 62% of them have 1 to 3 years of using experience, and 29.78% have 0.5 to 1 year experience. Regarding the most familiar LSP, 30.44% of middle-aged consumers choose the TikTok platform, 29.11% of them are familiar with the Kuaishou platform, and 23.56% are familiar with the Taobao Live platform.

**Table 3:** The basic information of respondents (*N* = 450)

Demographic variables	Category	Frequency	Percentage (%)
Gender	Female	229	50.89
	Male	221	49.11
Age	40–45	115	25.56
	46–50	147	32.67
	51–55	109	24.22
	56–60	79	17.56
Live shopping experience	0.5–1 year	134	29.78
	1–3 years	279	62
	Above 3 years	37	8.22
Most familiar platform	TiktTok	137	30.44
	Kuaishou	131	29.11
	Taobao Live	106	23.56
	Jingdong Live	54	12
	Others	22	4.89

The research utilises the variance-based structural equation modelling (SEM) and partial least squares (PLS) path modelling based on SmartPLS 2.0 for data analysis and model testing. The analysis of the measurement model and structural model is implemented on the SmartPLS 2.0, which is suitable for the research model study and meets the research aims (Chin et al. 2003; Chin 1998). Meanwhile, implementing PLS-SEM analysis on SmartPLS could provide a deep understanding of the research model, which previous scholars have long proved (Sarstedt and Cheah 2019; Hair et al. 2017).

### Common method bias

This study needs to test the common method bias because some correlations of the constructs are relatively high. To evaluate it, both the single-factor test and the measured latent-factor test are applied in this study (Podsakoff et al. 2003). Combined with the features of SmartPLS 2.0 and the research model, the study utilises the measured latent-factor test promoted by Liang et al. (2007). The average of trait factors explains 74.3% of the overall variance, and the average of method factors can explain 0.5% of the overall variance. The ratio between the average of trait factors and the average of method factors is 155.097, indicating that common method bias is not severe and the correlations of the constructs are acceptable (Liang et al. 2007). Meanwhile, the study also utilises the Harman single-factor test to check the issue of common method variance (CMV) (Zainol et al. 2018). Specifically, the Harman single-factor test is conducted by using principal component analysis and loading all items into a factor (Podsakoff et al. 2003). Through the data analysis, the single-factor model yields a total variance of 45.454%, which is less than the recommended threshold (Hapsari et al. 2020). Therefore, the common method bias is not serious.

### Measurement model

To test the research model, the study needs to analyse the reliability, convergent validity and discriminant validity. The Cronbach’s Alpha, composite reliability (CR), and average variance extracted (AVE) can be used by this research to evaluate the reliability (Hair et al. 1998). Based on the examination of the author Chin (1998), there are three criteria that need to be tested, including AVE greater than 0.50, CR higher than 0.70 and Cronbach’s Alpha greater than 0.70. Table 4 presents that all results meet the requirements and indicate good reliability.

**Table 4:** The results of AVE, CR, R Square and Cronbachs Alpha

	AVE	Composite reliability	Cronbachs alpha
CC	0.697	0.873	0.782
EU	0.701	0.876	0.787
IO	0.732	0.891	0.817
PP	0.693	0.871	0.779
SE	0.909	0.968	0.950
TB	0.722	0.886	0.808

*CC* counterfeiting concern, *SE* self-efficacy, *EU* ease of use, *IO* motivation to interact with online experts, *TB* trust building with online experts, *PP* purchase products recommended by online experts

The convergent validity and discriminant validity can be tested based on the confirmatory factor analysis. As the factor loadings and cross-loadings results show in Table 5, the markers’ loadings in each construct are highly correlated. To be specific, all marked constructs are considerably higher than other constructs, meaning the convergent validity and discriminant validity in this study are acceptable (Chin 1998; Wang et al. 2018). Meanwhile, based on Table 5, the range of marked items is from 0.781 to 0.958, significantly higher than 0.707, claiming that convergent validity is reasonable in this study (Ahmad et al. 2016; Joeliantina et al. 2019).

**Table 5:** Factor loadings and cross-loadings

	CC	EU	IO	PP	SE	TB
CC1	**0.804**	0.399	0.478	0.344	− 0.266	0.366
CC2	**0.869**	0.493	0.559	0.446	− 0.330	0.508
CC3	**0.830**	0.463	0.509	0.424	− 0.160	0.445
EU1	0.492	**0.856**	0.585	0.480	− 0.193	0.583
EU2	0.464	**0.836**	0.610	0.555	− 0.274	0.622
EU3	0.409	**0.820**	0.611	0.563	− 0.173	0.588
IO1	0.518	0.600	**0.847**	0.603	− 0.251	0.640
IO2	0.537	0.618	**0.849**	0.669	− 0.328	0.625
IO3	0.535	0.628	**0.871**	0.661	− 0.271	0.616
PP1	0.470	0.548	0.619	**0.781**	− 0.278	0.550
PP2	0.391	0.514	0.636	**0.856**	− 0.209	0.629
PP3	0.370	0.535	0.630	**0.859**	− 0.299	0.675
SE1	− 0.279	− 0.239	− 0.309	− 0.314	**0.958**	− 0.311
SE2	− 0.302	− 0.261	− 0.334	− 0.322	**0.955**	− 0.313
SE3	− 0.288	− 0.228	− 0.303	− 0.260	**0.948**	− 0.266
TB1	0.428	0.581	0.586	0.595	− 0.277	**0.834**
TB2	0.457	0.607	0.645	0.644	− 0.283	**0.847**
TB3	0.465	0.632	0.635	0.660	− 0.235	**0.867**

The Fornell-Larcker criterion can be used to examine the discriminant validity, and the AVEs’ square root on the diagonals can evaluate whether the discriminant validity is reasonable (Hwang and Lee 2012; Chin 1998; Fornell and Larcker 1981). As Table 6 states, the AVEs’ square root on the diagonals is significantly higher than other correlations, indicating that the discriminant validity meets the requirements. Meanwhile, the paper uses the assessment of cross-loading to measure the discriminant validity. Specifically, the value of cross-loading items should be higher than 0.5, and variables having the same representation must be distinguished from other factors with a similar representation and the same concept (Henseler et al. 2015). As Table 5 shows, cross-loading items are higher than 0.5, meeting the requirements.

**Table 6:** Correlations between constructs

	AVE	CC	EU	IO	PP	SE	TB
CC	0.697	**0.835**					
EU	0.701	0.543	**0.838**				
IO	0.732	0.619	0.720	**0.856**			
PP	0.693	0.487	0.637	0.753	**0.833**		
SE	0.909	− 0.304	− 0.255	− 0.331	− 0.314	**0.954**	
TB	0.722	0.530	0.714	0.733	0.746	− 0.311	**0.850**

The diagonals represent the square root of average variance extracted (AVE), and the lower cells represent the correlation among constructs

### Structural model

**Fig. 3:**
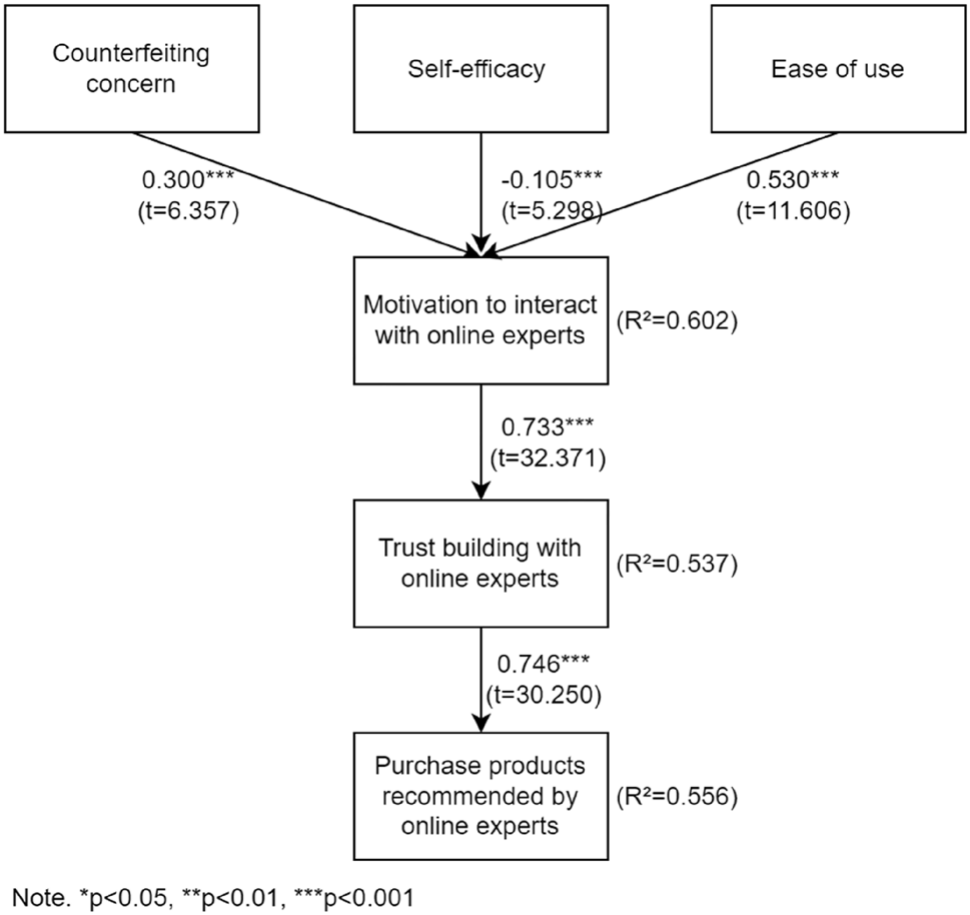
The path coefficients of the research model

The structural models can be tested by analysing the significance of the path coefficients and the t-statistical test of each path (Hair et al. 2016). After developing the bootstrapping on SmartPls 2.0, the results of path coefficients and the *t*-statistical test have been shown in Table 7. All hypotheses can be supported because *t*-statistics are remarkably higher than 1.96 (Hair et al. 2016). Meanwhile, *R*^2^’s values are higher than 0.4 (Fig. 3), providing evidence of acceptable reliability (McHaney et al. 2002).

**Table 7:** Hypotheses testing summary

	Original sample (O)	Standard error (STERR)	T statistics (|O/STERR|)	Hypothesis	Support?
CC → IO	0.300	0.047	6.357	H1+	Yes
EU → IO	0.530	0.046	11.606	H3+	Yes
IO → TB	0.733	0.023	32.371	H4+	Yes
SE → I0	− 0.105	0.020	5.298	H2−	Yes
TB → PP	0.746	0.025	30.250	H5+	Yes

According to the data analysis results presented in Table 7, the counterfeiting concern factor positively affects middle-aged Chinese online consumers’ motivation to interact with online experts (*β* = 0.300, *t* = 6.357, *p* < 0.001), supporting Hypothesis 1. Middle-aged online consumers’ self-efficacy negatively affects their motivation to interact with online experts (*β* = − 0.105, *t* = 5.298, *p* < 0.001), supporting Hypothesis 2. The ease of use of LSPs also positively influences middle-aged Chinese online consumers’ motivation to interact with online experts (*β* = 0.530, *t* = 11.606, *p* < 0.001), supporting Hypothesis 3. Meanwhile, the motivation to interact with online experts has a positive impact on middle-aged online consumers’ trust with them (*β* = 0.733, *t* = 32.371, *p* < 0.001), supporting Hypothesis 4, and the trust building with online experts also positively influences middle-aged Chinese online consumers to purchase the products recommended by online experts (*β* = 0.746, *t* = 30.250, *p* < 0.001), supporting Hypothesis 5.

## Discussion and implications

### Key findings

Based on the research results, several key findings can be presented. Firstly, the counterfeiting concern factor positively influences middle-aged Chinese online consumers’ motivation to interact with online experts. Considering middle-aged Chinese online consumers’ unique social and cultural backgrounds, they think that seeking advice from online experts is a reliable strategy to avoid the uncertain situation and prevent counterfeiting risks. Secondly, there is an apparent relationship between middle-aged online consumers’ self-efficacy and the motivation to interact with online experts. Given that many middle-aged online consumers lack comprehensive education, they are not familiar with live streaming shopping mode and tend to interact with online experts who can present professional certificates. Conversely, younger consumers who have high self-efficacy could rely on their judgements rather than online experts’ recommendations. Thirdly, the ease of use of LSPs positively affects middle-aged Chinese online consumers’ motivation to interact with online experts. This is because, different from traditional social media platforms, LSPs provide a comfortable environment for middle-aged online consumers and online experts. Various advanced functions, such as real-time video and group chat, benefit them to interact with online experts. Meanwhile, the motivation to interact with online experts positively affects middle-aged Chinese online consumers to build trust with them. This can explain why more and more online experts focus on their interactive skills, including marketing, advertising and communication skills. Finally, as hypothesis 5 shows, once online consumers build trust with online experts, they will be willing to purchase the recommended products. Hence, for online experts who want to boost product sales, interacting with online consumers and winning their trust are efficient marketing strategies to increase sales.

### Theoretical implications

Compared with previous studies, several theoretical implications are significant for future research. Firstly, regarding the unique social and cultural background, middle-aged online consumers from China are influenced significantly by traditional thinking characteristics, which can be reflected in the counterfeiting concern factor. To be specific, because of the traditional shopping experiences and conservative thinking way, middle-aged Chinese online consumers are more easily influenced by the counterfeiting concern factor than young consumer groups. Meanwhile, due to experiencing the difficult period when the economic system is unimproved, some middle-aged Chinese consumers prefer to think back and forth to avoid unnecessary financial trouble before making a final purchase decision. This explains why middle-aged Chinese online consumers tend to consult experts rather than make purchasing decisions alone. Thus, future study related to China’s social and cultural context needs to focus on the difference between different age groups and explore their shopping behaviours based on their particular backgrounds. Moreover, this paper applies the COM-B Behaviour Changing theory to analyse middle-aged Chinese online consumers’ specific live shopping behaviours, which could be the first application of this theory in consumer behaviour research. According to the COM-B research theory, the factors that affect individuals’ motivation can be divided into *Opportunity* and *Capability* units, which benefits researchers to explore consumer behaviours from macro and micro levels. Finally, this study combines the Emotional attachment theory with the COM-B Behaviour Changing model, and it claims the emotional factor would also significantly impact individuals’ motivation. Although existing literature refers to the Emotional attachment theory and applies the *Emotion* factor named counterfeiting concern to the study of online consumers’ shopping behaviour, none of them evaluates the counterfeiting concern impact on middle-aged Chinese online consumers. Thus, when the research is developed in unique social and cultural backgrounds, scholars need to consider the influence of the *Emotion* unit on individual thinking and behaviours. This updated theoretical framework has a significant contribution to future studies relevant to social and cultural climates on LSPs.

### Practical implications

With the popularity of online experts on China’s LSPs, this study is of great significance to enhance middle-aged online consumers’ live shopping awareness and improve the live streaming market environment. Specifically, most middle-aged Chinese online consumers have the motivation to interact with online experts because of the professional certificate displayed by experts. However, some experts might use their reputation for false advertising products, adversely impacting online consumers’ live shopping experience. Therefore, to enhance middle-aged Chinese online consumers’ live shopping awareness, it is necessary for related departments to change their thinking habits and encourage them to reassess the products’ quality. Besides enhancing middle-aged Chinese online consumers’ shopping awareness, this study also has a practical implication for online experts’ marketing strategies. As the data results present, the interaction with online consumers has a direct positive impact on the trust building between online consumers and online experts, driving online consumers to purchase products. Thus, from an online experts’ marketing perspective, if they aim to increase product sales constantly, they need to take advantage of their reputation and improve their interaction quality, including marketing and communication skills.

### Limitations and future study

Although this study contributes to future research related to live shopping analysis, there are still limitations that need to be improved in the prospective study. Firstly, the issues related to online experts have also happened in Western LSPs, but the findings of this research cannot be directly applied to Western LSPs’ research because of social and cultural background differences. Meanwhile, online consumers from different age groups, educational backgrounds and occupations could have different using preferences. This requires future studies to promote multi-group analysis based on these moderating factors. Therefore, in future research, scholars should not only focus on the comparison of online consumers between Eastern countries and Western countries but also develop a deep division of online consumer groups. Furthermore, based on the COM-B theory established by Michie et al. (2011), the *Opportunity* can be divided into physical and social opportunities. The ease of use is designed based on physical opportunity. Future studies will consider social opportunities, like related policies issued by local governments. Finally, considering that some middle-aged consumers lack online platform-using skills, it could hinder them from attending online surveys. Thus, in future studies, both online and offline surveys will be promoted, benefiting comprehensive data collection.

## Conclusion

In conclusion, most middle-aged Chinese online consumers prefer to communicate with online experts due to their particular social experience and cultural background. However, due to the absence of monitoring mechanisms on LSPs, some online experts could make false propaganda to increase their income. To solve this problem, this paper explores what factors would influence middle-aged Chinese online consumers to interact with online experts and purchase the products recommended by them. Based on the research model, the paper presents the process of trust building between middle-aged online consumers and online experts, and it also presents the impact of the emotional factor on online consumers’ live shopping behaviours. The paper aims to enhance online consumers’ live shopping awareness and improve the live streaming market environment by giving the relationships among different factors.
